# Phaseolin Attenuates Lipopolysaccharide-Induced Inflammation in RAW 264.7 Cells and Zebrafish

**DOI:** 10.3390/biomedicines9040420

**Published:** 2021-04-13

**Authors:** Su-Jung Hwang, Ye-Seul Song, Hyo-Jong Lee

**Affiliations:** School of Pharmacy, Sungkyunkwan University, 2066 Seobu-ro, Jangan-gu, Suwon 16419, Gyeonggi-do, Korea; sama3575@naver.com (S.-J.H.); summerai6533@naver.com (Y.-S.S.)

**Keywords:** Kushen, phaseolin, Radix *Sophorae flavescentis*, inflammation, zebrafish

## Abstract

Kushen (Radix *Sophorae flavescentis*) is used to treat ulcerative colitis, tumors, and pruritus. Recently, phaseolin, formononetin, matrine, luteolin, and quercetin, through a network pharmacology approach, were tentatively identified as five bioactive constituents responsible for the anti-inflammatory effects of *S. flavescentis*. However, the role of phaseolin (one of the primary components of *S. flavescentis*) in the direct regulation of inflammation and inflammatory processes is not well known. In this study, the beneficial role of phaseolin against inflammation was explored in lipopolysaccharide (LPS)-induced inflammation models of RAW 264.7 macrophages and zebrafish larvae. Phaseolin inhibited LPS-mediated production of nitric oxide (NO) and the expression of inducible nitric oxide synthase (iNOS), without affecting cell viability. In addition, phaseolin suppressed pro-inflammatory mediators such as cyclooxygenase 2 (COX-2), interleukin-1β (IL-1β), tumor necrosis factor α (TNF-α), monocyte chemoattractant protein-1 (MCP-1), and interleukin-6 (IL-6) in a dose-dependent manner. Furthermore, phaseolin reduced matrix metalloproteinase (MMP) activity as well as macrophage adhesion in vitro and the recruitment of leukocytes in vivo by downregulating Ninjurin 1 (Ninj1), an adhesion molecule. Finally, phaseolin inhibited the nuclear translocation of nuclear factor-kappa B (NF-κB). In view of the above, our results suggest that phaseolin could be a potential therapeutic candidate for the management of inflammation.

## 1. Introduction

Inflammation is a highly controlled, self-limiting process important for identifying and destroying exogenous pathogens in order to maintain homeostasis [1]. However, excessive inflammatory reactions are often the primary cause of chronic inflammation-related diseases such as Alzheimer’s disease, inflammatory bowel disease, type II diabetes, rheumatoid arthritis, cardiovascular diseases, and malignant tumors [2]. The early stages of inflammation fundamentally involve the infiltration of leukocytes into the lesion site [3]. Furthermore, when macrophages are activated by inflammatory stimuli such as endotoxins, they express adhesion molecules such as Ninjurin 1 (Ninj1) and produce pro-inflammatory factors such as nitric oxide (NO), tumor necrosis factor α (TNF-α), and interleukin-1β (IL-1β) through upregulation of nuclear factor-kappa B (NF-κB) [4]. Therefore, the inhibition of the activation and recruitment of leukocytes during the early stages of inflammation is considered a promising therapeutic strategy. The most widely used in vitro model for the development of anti-inflammatory agents is the murine macrophage-derived cell line RAW264.7, in which lipopolysaccharide (LPS) induces the expression of several inflammatory mediators and enhances the activity and migration of macrophages through NF-kB activation [5]. Here, we investigated the suppressive effect of phaseolin on inflammatory mediators, cell adhesion, and movement in LPS-stimulated RAW264.7 cells.

In Chinese traditional medicine, Kushen (Radix *Sophorae flavescentis*) has long been used for the prevention and/or treatment of various diseases such as ulcerative colitis [6] and pruritus [7]. In addition, Kushen has been widely used as an alternative treatment for cancer, and its components have demonstrated anti-tumorigenic effects against prostatic [8], gastric [9], and esophageal [10] cancer cell lines. However, the active components responsible for the anti-inflammatory effects of Kushen against ulcerative colitis or pruritus remain unclear. Recently, Chen et al. constructed a component–target–pathway network and tentatively identified five bioactive constituents (phaseolin, formononetin, matrine, luteolin, and quercetin) that were responsible for the anti-inflammatory effects of *S. flavescentis* [6]. Therefore, it is necessary to determine whether each of these five components represents anti-inflammatory activity and which mechanisms are involved. Among them, formonetin exhibits strong anti-inflammatory efficacy through inhibiting NF-κB signaling [11]. In addition, luteolin and quercetin possess a broad spectrum of anti-inflammatory activity [12,13]. However, the role of phaseolin, one of the primary components of Kushen, in the direct inhibition of inflammation and inflammatory processes has not yet been studied. Phaseolin, also known as abyssinone I or phaseollin, is a prenylated pterocarpan with the International Union of Pure and Applied Chemistry (IUPAC) name of 3,3-dimethyl-6b,12b-dihydro-3H,7H-furo [3,2-c:5,4-f’]dichromen-10-ol. In 1894, phaseolin was first identified from fungus-infected *Phaseolus vulgaris* (French bean) [14]. Phaseolin is a type of phytoalexin that is synthesized as a result of the plant’s response to pathogenic infections and has been shown to possess antimicrobial activity against *Staphylococcus aureus* and *Mycobacterium smegmatis* [15], as well as anti-oxidant efficacy [16]. Besides its anti-fungal effect [14], phaseolin exhibits anti-neoplastic effects by inducing apoptotic cell death in hepatoma cells [17]. In this study, therefore, we investigated the beneficial role of phaseolin on the endotoxin-induced inflammatory models.

Since the zebrafish (*Danio rerio*) is small in size, has a similar anatomy and physiology to humans, and possesses both innate and adaptive immunity, it has been regarded as a useful tool to investigate the efficacy, toxicity, and mode of action of drug candidates in vivo [18]. In addition, zebrafish larvae remain almost transparent for 24 h after fertilization, allowing for easy observation inside without further processing [19]. Therefore, it is possible to easily observe cell-specific movements and the numerical changes of cells in zebrafish by cell-specific markers or dyes in vivo. For example, staining with neutral red or using CD11b-specific antibodies leads to easy identification of macrophage migration and quantitative changes in zebrafish larvae [20,21]. Recently, the use of zebrafish larvae as inflammatory models has increased significantly; in this process, the zebrafish larvae are exposed to endotoxin by immersion techniques or microinjection into the yolk, and exhibit inflammatory responses similar to those of mammals [22]. Here, we examined the inhibitory effect of phaseolin on the mobility of macrophages and neutrophils in zebrafish embryos exposed to LPS.

In this study, we investigated the role of phaseolin in lipopolysaccharide (LPS)-induced inflammation models in vivo and in vitro, and revealed the detailed mechanisms underlying its pharmacological effects. We demonstrated that phaseolin exerts its anti-inflammatory activities in RAW264.7 cells as well as zebrafish larvae at least in part by downregulating NF-κB signaling components. Consequently, our findings suggest that phaseolin is a potential therapeutic agent which targets the NF-κB pathway for the treatment of inflammation.

## 2. Materials and Methods

### 2.1. Cell Culture and Reagents

The RAW 264.7 murine macrophage cells and HL-60 human promyelocytic leukemia cells were purchased from the Korean Cell Line Bank. RAW 264.7 cells were grown in Dulbecco’s modified Eagle’s medium (DMEM, Gibco BRL, Gaithersburg, MD, USA) supplemented with 10% fetal bovine serum (FBS, Gibco BRL). HL-60 cells were incubated under the same conditions using RPMI 1640 medium instead of DMEM. In order to induce differentiation into the neutrophil-like cells, HL-60 cells were incubated with 1.25% *v/v* DMSO for 5 days without media change. Lipopolysaccharide, gelatin, fibronectin, type I collagen, laminin, Sudan black, neutral red, and Griess reagent were purchased from Sigma-Aldrich (St. Louis, MO, USA). Phaseolin was purchased from Molport (CAS: 13401-40-6, Beacon, NY, USA). Polyclonal antibodies against inducible nitric oxide synthase (iNOS), tubulin, nuclear factor kappa B (NF-κB), inhibitor kappa B (I-κB), glyceraldehyde 3-phosphate dehydrogenas (GAPDH), and lamin A were purchased from Santa Cruz Biotechnology. Polyclonal antibodies against endogenous Ninj1 were obtained from Dr. Kyu-Won Kim (Seoul National University, Seoul, Korea).

### 2.2. Zebrafish Maintenance, Toxicity Test, and Induction of Endotoxin Shock

Zebrafish (*Danio rerio*, 4–6 month-old AB line) of both sexes were purchased commercially and maintained at 28.5 °C with a 14:10 h light/dark cycle in a recirculating tank system using local tap water (pH 7.2–7.6). The handling and care of adult zebrafish were approved by the Institutional Animal Care and Use Committee (IACUC) of Sungkyunkwan University in Suwon, South Korea. All the experiments were conducted with an effort to minimize the number of animals and reduce the deleterious effects on them that were bound to be caused by the procedures employed.

To evaluate the developmental toxicity and survival rate, zebrafish embryos at 24-h-post fertilization (24 hpf) were treated with phaseolin at 27 °C. After 24 h (48 hpf), embryos were placed in a fresh E3 solution and peeled using a needle or tweezers. Then, the heart rate was measured by observing the phenotype and counting the heart beats under a microscope.

The endotoxin-induced zebrafish inflammation model was prepared as previously described [23,24]. Briefly, larvae at 3 days post-fertilization (dpf) were incubated with embryo media containing vehicle or 50 or 100 μM phaseolin at 28.5 °C. After 18 h of incubation, 2 nL of LPS (1.0 mg/mL) were injected into the yolks of each larva using a pneumatic microinjector (World Precision Instruments, Cambridge, MA, USA). After LPS stimulation, neutrophils and macrophages were tracked by Sudan black and neutral red assays, respectively.

### 2.3. Endotoxin-Induced Uveitis in Mouse

The endotoxin-induced uveitis mouse model was prepared as previously described [25], except with regard to the administration schedule. Briefly, mice were injected intraperitoneally with a dose of 330 μg/kg of LPS. At 2 and 12 h after LPS injection, topical eye drops (5 μL/eye) containing either vehicle, 0.01% phaseolin, or 0.1% phaseolin were administered twice a day. The schedule was repeated for three days. The mice were sacrificed 24 h after the final injection of LPS. The handling and care of the animals were approved by the Institutional Animal Care and Use Committee (IACUC) of Sungkyunkwan University in Suwon, South Korea.

### 2.4. Nitric Oxide (NO) Assay

The quantity of NO in the culture supernatant was measured using Griess reagent. Briefly, RAW 264.7 cells were pre-incubated with 50 or 100 μM of phaseolin for 1 h and then treated with LPS (1 μg/mL) for 24 h. The supernatant was then incubated with Griess reagent for 10 min and the absorbance was measured at 540 nm. Fresh culture medium was used as a blank.

### 2.5. Cell Viability Test

The RAW 264.7 cells were seeded at 4 × 10^3^ cells per well in a 96-well plate and treated with various concentrations of phaseolin (ranging from 31.25–500 μM) for 24 h. After the treatment, the culture media were removed and fresh media with 10 μL of 3-(4,5-Dimethylthiazol-2-yl)-2,5-diphenyltetrazolium bromide (MTT) solution (Promega, Mandison, WI, USA) was added to each well and incubated for 2 h at 37 °C. The purple formazan crystal was dissolved in dimethyl sulfoxide and the absorbance was measured at 490 nm. The cytotoxicity of RAW 264.7 cells was calculated relative to the untreated cells. For HL-60 cells, 3-(4,5-dimethylthiazol-2-yl)-5-(3-carboxymethoxyphenyl)-2-(4-sulfophenyl)-2H-tetrazolium (MTS) was used instead of MTT.

### 2.6. RNA Isolation and Reverse Transcription-Polymerase Chain Reaction (RT-PCR)

Total RNA was isolated using TRIzol reagent (Invitrogen, Waltham, MA, USA) and reverse-transcribed to complementary DNA (cDNA) using the M-MLV Reverse Transcriptase kit (Promega). Real-time PCR was performed on a Rotor-Gene Q cycler (Qiagen, Venlo, The Netherlands) using the Rotor Gene SYBR Green RT-PCR kit (Qiagen) following the manufacturer’s instructions. The PCR primers were purchased from Bioneer and are as follows: iNOS (forward, 5′-cagctgggctgtacaaacctt-3′; reverse 5′-cattggaagtgaagcgtttcg-3′); interleukin-1β (IL-1β, forward, 5′-aagggctgcttccaaacctttgac-3′; reverse, 5′-tgcctgaagctcttgttgatgtgc-3′); TNF-α (forward, 5′- catcttctcaaaattcgagtgacaa-3′; reverse, 5′- tgggagtagacaaggtacaaccc-3′); cyclooxygenase 2 (COX-2, forward, 5′-ttcaaaagaagtgctggaaaaggt-3′; reverse, 5′-gatcatctctacctgagtgtcttt-3′); IL-6 (forward, 5′-gaggataccactcccaacagacc-3′; reverse, 5′-aagtgcatcatcgttgttcataca-3′); monocyte chemoattractant protein-1 (MCP-1, forward, 5′-cttctgggcctgctgttca-3′; reverse, 5′-ccagcctactcattgggatca-3′); chemokine (C-X-C motif) ligand 1 (CXCL1, forward, 5′-tggggacaccttttagcatc-3′; reverse, 5′-cttgaaggtgttgccctc-3′); IL-4, forward, 5′-ggtctcaacccccagctagt-3′; reverse, 5′-gccgatgatctctctcaagtgat-3′); IL-10 (forward, 5′-gcgctgtcatcgatttctcc-3′; reverse, 5′-atggccttgtagacaccttgg-3′); IL-13 (forward, 5′-cctggctcttgcttgcctt-3′; reverse, 5′-ggtcttgtgtgatgttgctca-3′); β-actin (forward, 5′-agagggaaatcgtgcgtgac-3′; reverse 5′-ggccgtcaggcagctcatag-3′). β-actin was used as an internal control. PCR-amplified products were separated on 1.5% agarose gels and visualized by RedSafe nucleic acid staining (IntRON Biotechnology, Seongnam, Korea) and UV irradiation.

### 2.7. Protein Extract and Western Blotting

A total of 50 × 10^6^ RAW 264.7 cells were cultured in 100-mm dishes without or with varying concentrations of phaseolin for 24 h in the presence of LPS. Following treatment, cells were harvested and lysed in lysis buffer (0.5% Triton, 50 mM b-glycerophosphate, pH 7.2, 0.1 mM sodium vanadate, 2 mM MgCl_2_, 1 mM ethylene glycol tetraacetic acid, 1 mM dithiothreitol, 0.1 mM phenylmethylsulfonyl urea, 2 μg/mL leupeptin, and 4 μg/mL aprotinin). Proteins were resolved by 12% sodium dodecyl sulfate-polyacrylamide gel electrophoresis (SDS-PAGE) and transferred to nitrocellulose membranes. The membranes were incubated with primary antibodies at 4 °C for 18 h, washed, and then incubated with the secondary antibodies at room temperature for 1 h. The protein bands were detected by chemiluminescence (FUSION-SL4, Vilber Lourmat, Collegien, Marne-la-Vallée, France).

### 2.8. ELISA

The protein levels of IL-6, TNF-α, and IL-10 were determined using ELISA kits according to the manufacturer’s protocol. A mouse IL-6 (catalog number BSKM1004), TNF-α (catalog number BSKM1002), and IL-10 (catalog number BSKM1007) kits were purchased from Bioss (Woburn, MA, USA).

### 2.9. Cell Adhesion Assay

The wells in the 96-well plate were coated with four different types of extracellular matrix (ECM) proteins, fibronectin, type I collagen, laminin, and gelatin. RAW 264.7 cells were incubated in ECM protein-coated wells for 15 min. After washing with phosphate-buffered saline, the attached cells were stained with crystal violet and then washed thrice. Following washing, the cells were lysed with 0.2% NP-40, and the absorbance of lysates was measured at 590 nm.

### 2.10. Cell Migration Assay

RAW 264.7 cells and differentiated HL-60 (dHL-60) cells were added to the upper chamber of each Transwell (Corning Inc., Corning, NY, USA) at 1 × 10^5^ cells per well. The lower chamber was filled with a medium supplemented with 10% FBS, and after 4 h the cells on the upper surface of the membrane were removed with swabs. Next, the cells on the lower side were fixed with 37% paraformaldehyde and stained with crystal violet. The number of migrated cells was counted under an Eclipse Ts2 microscope (Nikon, Tokyo, Japan).

### 2.11. Immunofluorescence Microscopy and Quantification

RAW 264.7 cells were plated at 5 × 10^4^ cells per well in 8-well chamber slides (Falcon, San Jose, CA, USA) with or without phaseolin for 24 h in the presence of LPS, and then fixed in 4% paraformaldehyde for 10 min. Following permeabilization with 0.1% Triton X-100 for 15 min, the cells were blocked with 10% normal goat serum for 30 min and incubated with primary antibodies at 1:500 dilution for 18 h at 4 °C. After washing, the cells were incubated with Alexa488-conjugated IgG (Molecular Probes) at 1:1000 dilution for 50 min, and nuclear staining was performed using 4′-6-diamidino-2-phenylindole (DAPI, Invitrogen). Alexa488 was excited with a 488-nm laser (110mW) and emission was filtered by a 525/50 nm. To avoid zero signals or saturation, the gain was adjusted to achieve a mean image intensity of about 180 (out of 256 grey levels). Images were captured using an Eclipse Ts2 microscope (Nikon, Japan) and the pixel intensities of iNOS and Ninj1 were measured as the percent area of immunoreactivity using ImageJ (NIH, Bethesda, MD, USA).

### 2.12. Gelatin Zymography

RAW 264.7 cells were plated at 5 × 10^6^ cells per 60-mm dish and incubated with or without phaseolin for 24 h in the presence of LPS. The culture supernatant was resolved using 10% SDS-PAGE containing 1.5 mg/mL gelatin. Following electrophoresis, the gel was washed with 2.5% Triton X-100, 50 mM Tris-HCl (pH 7.5), and then incubated overnight at 37 °C in a buffer containing 150 mM NaCl, 5 mM CaCl_2_, and 50 mM Tris-HCl (pH 7.6). The gel was subsequently stained with 0.1% Coomassie Brilliant Blue R-250, and destained in 10% methanol and 5% glacial acetic acid to reveal zones with gelatinase activity. All zymography experiments were performed in triplicate.

### 2.13. Statistical Analysis

All the data are represented as average (±standard deviation) of triplicate experiments and were changed to relative values. Statistical analyses were performed using GraphPad Prism 6 (San Diego, CA, USA) and the differences between treatment and control groups were evaluated by Student’s *t*-test or one-way ANOVA followed by a post-hoc test. A *p*-value of <0.05 was considered to be statistically significant.

## 3. Results

### 3.1. Phaseolin Inhibited NO Production and iNOS Expression in LPS-Stimulated RAW 264.7 Macrophages without Affecting Cell Viability

**Figure 1:**
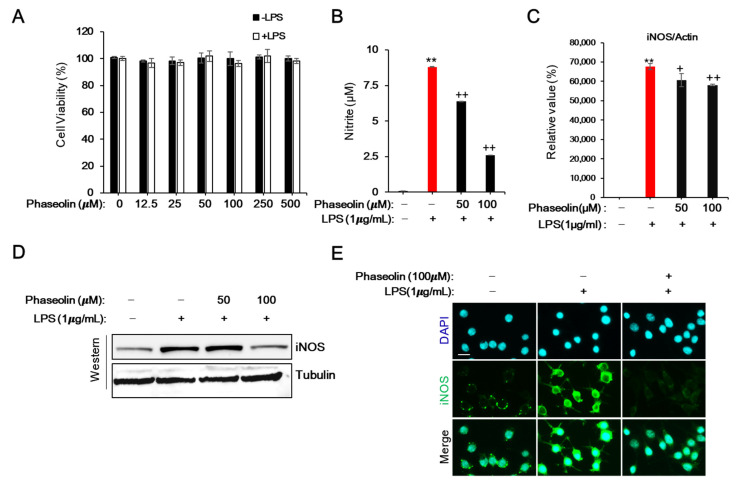
Effects of phaseolin on cell viability, nitric oxide (NO) production, and inducible nitric oxide synthase (iNOS) expression in lipopolysaccharide (LPS)-stimulated RAW 264.7 macrophages. Cell viability (**A**) was assessed using -(4,5-Dimethylthiazol-2-yl)-2,5-diphenyltetrazolium bromide (MTT) reduction assay and is expressed as the percentage of live cells over the control cells. The NO assay (**B**) was performed using the Griess reagent. Values represent the mean ± SD of 3 independent experiments. ** *p* < 0.01, vehicle vs. LPS-treated group; + *p* < 0.05 and ++ *p* < 0.01, LPS vs. LPS + phaseolin-treated group. Expression of iNOS at the mRNA level (**C**) was measured using real-time PCR. Expression of iNOS at the protein level (**D**) was measured using Western blotting. Representative immunofluorescence images (**E**) using anti-iNOS antibody (green). DAPI (4′,6-diamidino-2-phenylindole) was used for nuclear staining (blue). A scale bar indicates 10 μm.

To determine the optimal concentration of phaseolin that does not induce cellular toxicity, RAW 264.7 cells were treated with various concentrations of phaseolin for 24 h, and then the cell viability was measured using tetrazolium-based colorimetric assay. The MTT assay showed that phaseolin at a concentration of 100 μM had no toxicity; however, treatment with 250 μM phaseolin slightly reduced the cell viability (Figure 1A). Hence, 100 μM of phaseolin were used for subsequent experiments. Further, to investigate the effect of phaseolin on NO production during LPS-induced inflammation, the cells were pre-treated with phaseolin for 1 h, followed by LPS stimulation for 24 h. The results demonstrated that phaseolin significantly inhibited LPS-induced NO production in a dose-dependent manner (Figure 1B). Next, we investigated whether the reduction in NO production by phaseolin was due to the decreased expression of its synthesizing enzyme, iNOS. As expected, phaseolin markedly decreased the LPS-induced expression of iNOS at both the mRNA and protein levels (Figure 1C,D). The reduction in iNOS level by phaseolin was further confirmed by immunofluorescence staining (Figure 1E). Together, these results suggest that phaseolin inhibits LPS-induced NO production and iNOS expression in RAW 264.7 cells without affecting cell viability.

### 3.2. Phaseolin Suppressed Pro-Inflammatory Cytokines and Other Inflammation-Related Genes in LPS-Stimulated RAW 264.7 Cells

**Figure 2:**
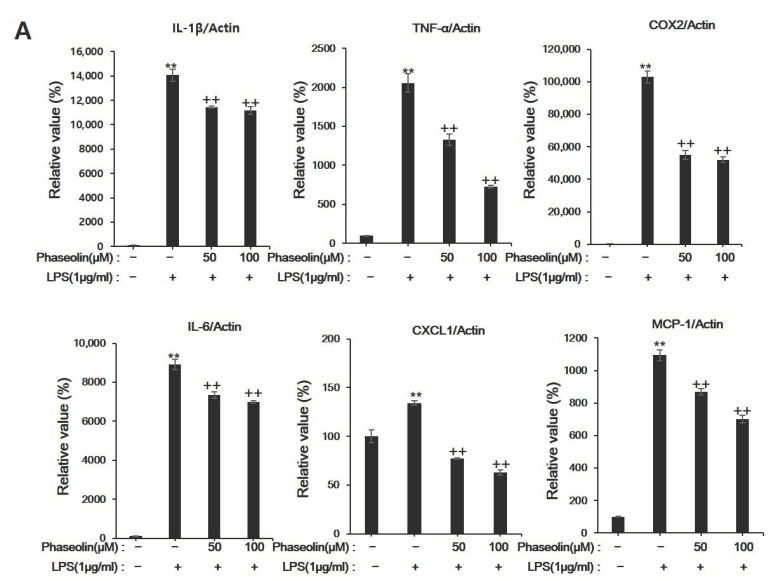

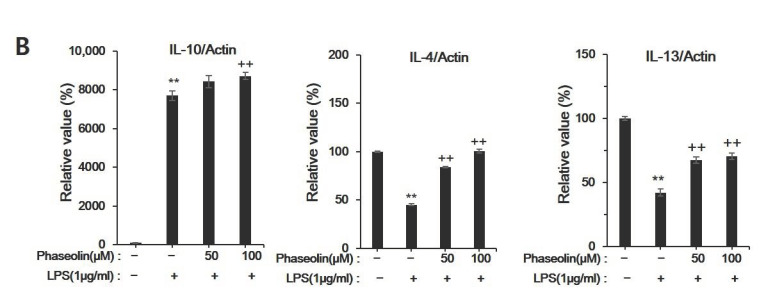

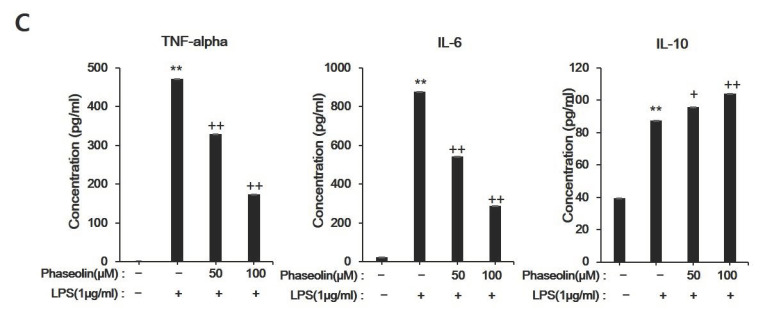
Effects of phaseolin on the mRNA expression levels of pro-inflammatory cytokines and other inflammation-related genes. Expression of (**A**) pro-inflammatory genes (such as interleukin (IL)-1β and tumor necrosis factor-α) and (**B**) anti-inflammatory mediators (such as IL-4, IL-10, and IL-13) at the mRNA level upon phaseolin treatment and LPS stimulation. The mRNA expression in the treated groups was normalized to that of in the negative control group (considered as 1) and is indicated as a relative value. ** *p* < 0.01, vehicle vs. LPS-treated group; + *p* < 0.05 and ++ *p* < 0.01, LPS vs. LPS + phaseolin-treated group. (**C**) Protein expression levels of pro-inflammatory genes (IL-6 and TNF-α) and an anti-inflammatory mediator (IL-10) upon phaseolin treatment and LPS stimulation. IL: interleukin.

Next, we determined the effects of phaseolin on pro-inflammatory cytokines and other inflammatory factors, including interluekin (IL)-1β and TNF-α. LPS stimulation increased the mRNA expression levels of IL-1β and TNF-α, whereas phaseolin effectively inhibited LPS-induced expression of IL-1β and TNF-α (Figure 2A). In order to further verify the inhibitory effects of phaseolin, the expression of other inflammation-related genes was investigated. Furthermore, phaseolin reduced the LPS-induced mRNA expression of COX-2, IL-6, CXCL1, and MCP-1. We further analyzed the changes of anti-inflammatory mediators such as IL-4, IL-10, and IL-13. When treated with LPS, the expression levels of IL-4, IL-10, and IL-13 were decreased compared to control, and when phaseolin was co-treated with LPS, this reduction was reversed (Figure 2B). In addition, we tried to confirm whether phaseolin affected the protein expression level of pro-inflammatory and anti-inflammatory mediators in RAW 264.7 cells. As shown in Figure 2C, phaseolin notably inhibited the increase in protein levels of IL-6, TNF-α, IL-4, and IL-10 induced by LPS stimulation. Hence, these data suggest that phaseolin may decrease the expression levels of pro-inflammatory cytokines and chemokines during inflammation.

### 3.3. Phaseolin Suppressed Cell-ECM Adhesion by Downregulating Ninj1

**Figure 3:**
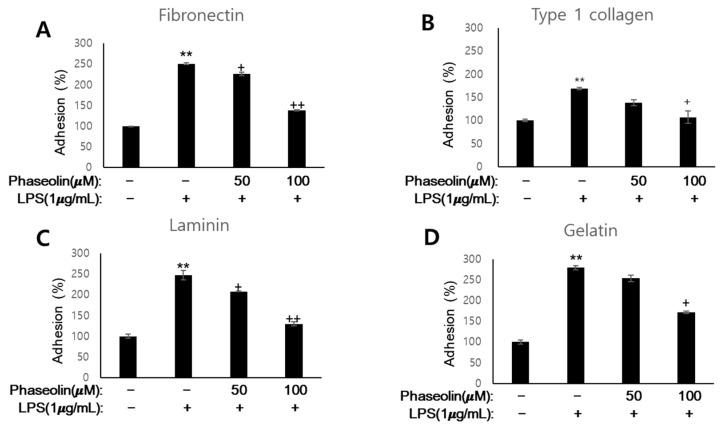
Effects of phaseolin on cell-to-matrix adhesion, in vitro. RAW 264.7 cells were treated with LPS (1 μg/mL) alone or LPS + phaseolin (50 or 100 μM) for 24 h and then incubated in extracellular matrix protein-coated 96-well plates for 15 min. Adhesion to fibronectin (**A**), type I collagen (**B**), laminin (**C**), and gelatin (**D**) was quantified, expressed as relative values, and normalized to 100%. Values shown in the graphs indicate the mean ± SD. ** *p* < 0.01, vehicle vs. LPS-treated group; + *p* < 0.05 and ++ *p* < 0.01, LPS vs. LPS plus phaseolin-treated group.

**Figure 4:**
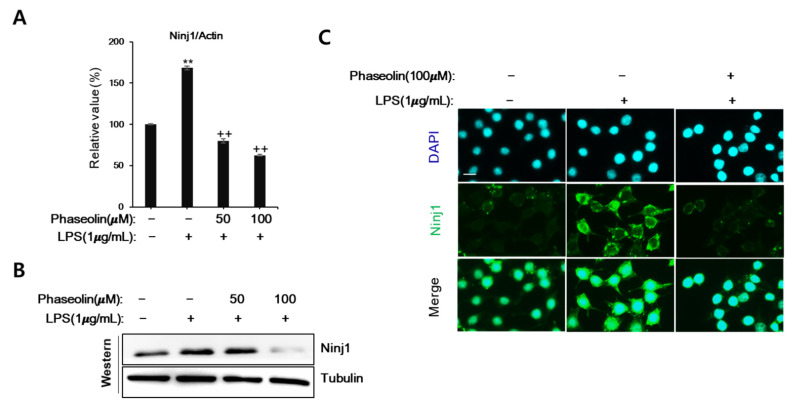
Effects of phaseolin on Ninjurin1 (Ninj1) expression, in vitro. RAW 264.7 cells were treated with LPS (1 μg/mL) alone or LPS + phaseolin (50 or 100 μM) for 24 h and then Ninj1 expression was assessed using real-time polymerase chain reaction (PCR) (**A**) and Western blotting (**B**). (**C**) Immunofluorescence staining of Ninj1 (green). Nuclei were stained with 1 μg/mL DAPI (blue). A scale bar indicates 10 μm. ** *p* < 0.01, vehicle vs. LPS-treated group; ++ *p* < 0.01, LPS vs. LPS + phaseolin-treated group.

During the early inflammatory phase, cytokines increase the adhesiveness leukocytes to the extracellular matrix (ECM) proteins near the sites of inflammation [26]. Therefore, we examined the effect of phaseolin on the adhesion of macrophages to ECM components including fibronectin, type I collagen, laminin, and gelatin, in vitro. When activated by LPS, RAW 264.7 cells showed increased adhesion to all the four ECM proteins. However, phaseolin significantly suppressed LPS-mediated increase in cell-to-matrix adhesion (Figure 3). We further analyzed the mode of loss of cell adhesion caused by phaseolin. Cell adhesion molecules are essential for cell–cell interaction as well as cell-to-matrix adhesion during leukocyte homing. Ninj1, also known as nerve-injury induced protein 1, is one of the key molecules involved in leukocyte recruitment through cell adhesion and movement [27]. Phaseolin abolished the LPS-induced increase in Ninj1 mRNA and protein expression (Figure 4A,B). The inhibitory effect of phaseolin on Ninj1 protein expression was further confirmed by immunofluorescence staining (Figure 4C). Together, these findings indicate that the inhibitory effect of phaseolin may be associated with reduced cell-to-matrix adhesion through Ninj1 downregulation.

### 3.4. Phaseolin Inhibited Cell Migration and Matrix Metalloproteinase (MMP)

**Figure 5:**
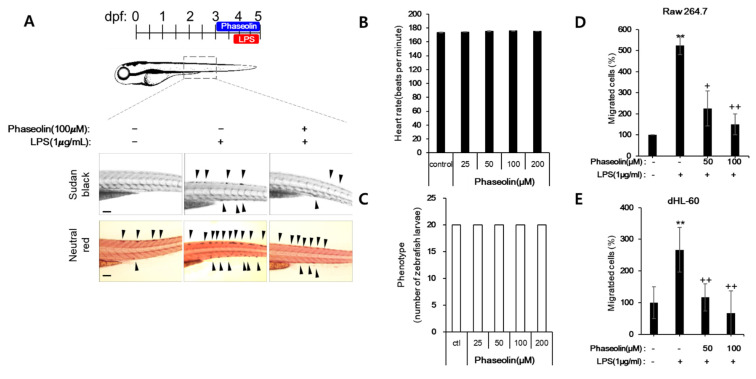
Effects of phaseolin on migration of neutrophils and macrophages, in vivo and in vitro. The representative pictures of neutrophils (upper, black) and macrophages (lower, red) are shown (**A**). Zebrafish larvae (3 days post fertilization, dpf) were pretreated with phaseolin (50 or 100 μM) for 18 h and then injected with LPS (1 μg/mL). After 30 h, zebrafish larvae were stained with Sudan black to detect neutrophil recruitment or neutral red to detect macrophage migration. Arrowhead indicates the migrating cell. Heart toxicity of phaseolin (**B**) and phenotype distribution after phaseolin treatment (**C**). Zebrafish larvae were exposed to different concentrations (0.125–2 mM) of phaseolin between 24 h post fertilization (hpf) and 48 hpf. The heart rate was measured by observing heartbeat under a microscope. The developmental defects were assessed by phenotype distribution such as survival and morphological changes using a stereomicroscope coupled with a digital camera (Nikon). TD: trunk deformity; H: hemorrhage; NT: normal trunk. The migration of macrophage-like RAW 264.7 cells (**D**) and neutrophil-like differentiated HL-60 (dHL-60) cells (**E**) was analyzed using transwell migration assays. ** *p* < 0.01, vehicle vs. LPS-treated group; + *p* < 0.05 and ++ *p* < 0.01, LPS vs. LPS plus phaseolin-treated group. A scale bar indicates 0.1 mm.

**Figure 6:**
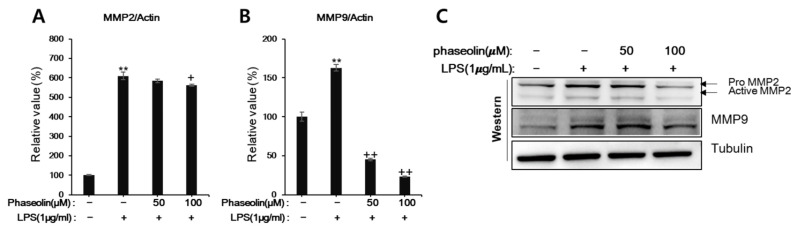
Effects of phaseolin on matrix metalloproteinase (MMP)-9 and MMP-2 expression. RAW 264.7 cells were treated with LPS (1 μg/mL) alone or LPS + phaseolin (50 or 100 μM) for 24 h and then MMP-9 and MMP-2 expression was assessed by realtime-PCR (**A**,**B**) and Western blotting (**C**). ** *p* < 0.01, vehicle vs. LPS-treated group; + *p* < 0.05 and ++ *p* < 0.01, LPS vs. LPS plus phaseolin-treated group.

Macrophages and neutrophils play critical roles in innate immune response, and their migration into the inflamed tissues is crucial in order to respond to infections or inflammatory stimuli [28,29]. Thus, we examined whether phaseolin affected the migration of macrophages and neutrophils in LPS-stimulated zebrafish in vivo. As shown in Figure 5A, phaseolin decreased the numbers of neutrophils and macrophages, which were elevated after LPS stimulation. To examine whether the inhibitory effect of phaseolin on cell migration resulted from its toxicity, the heart rate and phenotype distribution were analyzed using zebrafish larvae. Up to 200 μM of phaseolin, zebrafish showed a normal heartbeat rate and normal phenotype without death and hemorrhages (Figure 5B,C). Next, we tried to confirm the suppressive effect of phaseolin on the migration of neutrophil and macrophage using neutrophil-like differentiated HL-60 (dHL-60) cells and RAW 264.7 cells. As shown Figure 5D,E, LPS treatment increased the migration of both dHL-60 and RAW 264.7 cells, and phaseolin attenuated the increase of cell migration induced by LPS stimulation. We further analyzed the mode of loss of cell migration caused by phaseolin. As the active matrix metalloproteinases MMP-9 and MMP-2 are required for promoting the migration of leukocytes, the effect of phaseolin on MMP-9 and MMP-2 expression was assessed. When stimulated by LPS, the mRNA and protein expression of MMP-9 and MMP-2 increased; however, phaseolin significantly reduced the LPS-mediated increase in their expression in vitro (Figure 6). Together, these results suggest that the anti-inflammatory effects of phaseolin may be associated with reduced cell migration through downregulation of MMPs.

### 3.5. Phaseolin Inhibited NF-κB Translocation In Vitro

**Figure 7:**
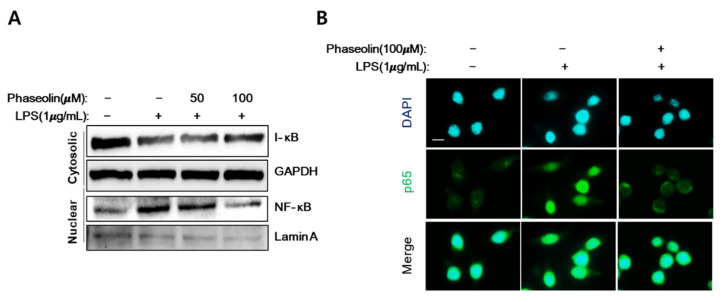
Effects of phaseolin on nuclear NF-κB translocation and inhibitor kappa B (I-κB) degradation. RAW 264.7 cells were treated with LPS (1 μg/mL) alone or LPS + phaseolin (50 or 100 μM) for 24 h and then the expression levels of nuclear NF-κB and cytoplasmic I-κB were assessed using Western blotting (**A**) and immunofluorescence staining of NF-κB (**B**, green). Nuclei were stained with 1 μg/mL DAPI (blue). A scale bar indicates 10 μm.

During inflammation, NF-κB is activated and translocated into the nucleus to activate its target genes, including pro-inflammatory mediators such as iNOS, COX-2, and IL-1 [29]. When activated with LPS, a marked increase in the accumulation of nuclear NF-κB and degradation of cytoplasmic inhibitor kappa B (I-κB) were observed. Phaseolin decreased the NF-κB accumulation in the nucleus and I-κB breakdown in the cytoplasm, similar to the levels observed in the control group (Figure 7A). The inhibitory effects of phaseolin on nuclear NF-κB accumulation was further confirmed by immunofluorescence staining (Figure 7B). These data indicate that the anti-inflammatory effect of phaseolin is associated with reduced translocation of NF-κB.

### 3.6. Phaseolin Attenuated Endotoxin-Induced Uveitis in Mice

**Figure 8:**
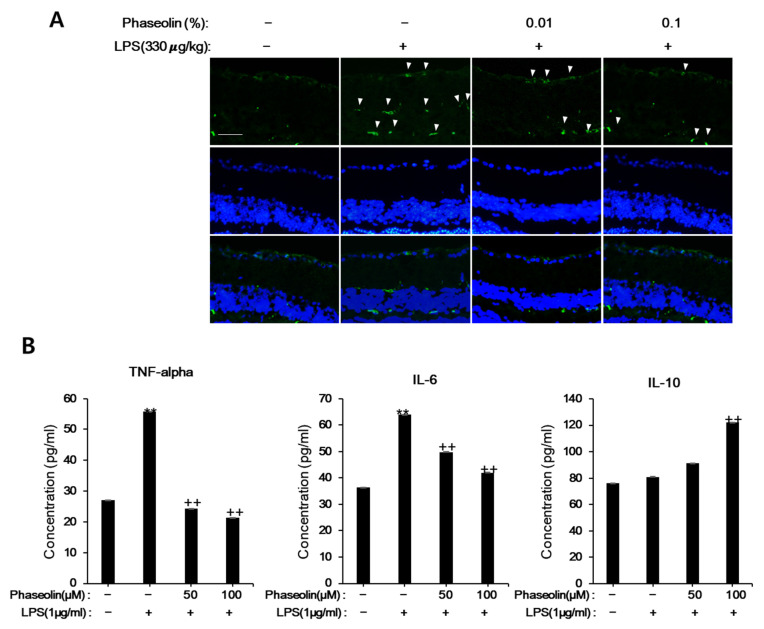
Effects of phaseolin on Ninj1 expression in LPS-induced uveitis mouse model. Mice were injected intraperitoneally (i.p.) with LPS (330 μg/kg), followed by eye drops of either vehicle or 0.01% or 0.1% phaseolin twice a day for 3 days. Twenty-four hours after the last LPS treatment, the mice were sacrificed and enucleated eyes were subjected to immunofluorescent staining (**A**) and ELISA (**B**). In the immunofluorescence staining of Ninj1, the nuclei were stained with 1 μg/mL DAPI (blue). ** *p* < 0.01, vehicle vs. LPS-treated group; ++ *p* < 0.01, LPS vs. LPS + phaseolin-treated group. A scale bar indicates 50 μm.

Next, we examined whether phaseolin exhibited anti-inflammatory effect in vivo, we used the endotoxin-induced uveitis mouse model, in which phaseolin was treated after LPS stimulation. As previously reported, LPS increased the recruitment of Ninj1-expressing macrophages into the retina. Phaseolin notably attenuated the increase of cell infiltration that was increased by LPS stimulation (Figure 8A). In order to further confirm the inhibitory effects of phaseolin on LPS-induced inflammation, the protein levels of TNF-α, IL-6, and IL-10 were analyzed. As expected, phaseolin reduced the increase in IL-6 and TNF-α that was induced by endotoxin and increased the expression level of IL-10 (Figure 8B). These data suggest the therapeutic role of phaseolin under endotoxin-induced retinal inflammation.

## 4. Discussion

Phytoalexin is a secondary metabolite of plants and accumulates in response to diverse stress conditions including ultraviolet rays, freezing, wounds, and pathogens [30]. It has been found in at least 70 plant species, such as potato, rice, tomato, and soybean [31]. It consists of a number of components with diverse structures, including stilbenes, flavonoids, and isoprenoids [32], which play important roles in the plant defense system against bacterial and fungal infections [33]. In addition, phytoalexin displays beneficial roles in humans, such as anti-inflammatory and anti-neoplastic effects [31,34]. For instances, resveratrol, as a representative phytoalexin, exhibits wide range of health-promoting effects, including neuroprotective, anti-tumor, and cardioprotective effects [35]. Hence, researchers are interested in exploring the structure, function, and mode of action of phytoalexin.

Phaseolin, a phytoalexin, possesses anti-microbial activity against *Staphylococcus aureus* [15], as well as anti-tumorigenic activity against H4IIE hepatoma cells [17]. Apart from these two reports, little research has been done on what biological activity phaseolin represents and what molecular mechanisms work. Recently, it has been reported that Kushen (Radix *Sophorae flavescentis*), which is a promising herbal medicine against inflammation, contains phaseolin as one of the bioactive ingredients [36]. In addition, Chen et al. predicted that phaseolin is as a major bioactive ingredient responsible for the anti-inflammatory activity represented by Kushen based on a component–target–pathway network [6]. However, the mechanisms underlying the anti-inflammatory effects of Kushen and the biological relevance of its component phaseolin against inflammation are largely unknown. This is the first study to report the anti-inflammatory action of phaseolin in vitro and in vivo using LPS-induced inflammation models of RAW 264.7 cells, zebrafish, and mouse uveitis, respectively. Our results suggested that phaseolin (1) reduced NO synthesis and iNOS expression, (2) suppressed pro-inflammatory mediators and increased anti-inflammatory mediators, (3) inhibited cell-to-ECM adhesion through Ninj1 downregulation, (4) reduced cell migration, and MMP expression, and (5) reduced nuclear NF-κB translocation. The inhibitory effect of phaseolin on IL-6 expression was consistent with Chen’s expectations through a network pharmacology analysis [6].

NF-κB is regarded as a master regulator of inflammation in several diseases such as inflammatory bowel diseases, atherosclerosis, and rheumatoid arthritis [37]. Because NF-κB plays an important role in the survival, activation, or differentiation of innate immune cells, as well as in inducing the expression of pro-inflammatory mediators including cytokines and chemokines, there is still a significant demand for effective inhibitors to NF-κB [38]. In addition, it has been reported that dysregulated NF-κB causes tumor formation, metastasis, and chemoresistance [39]. Here we found that phaseolin inhibited nuclear translocation of NF-κB and decreased the adhesion and migration of macrophages by downregulation of Ninj1. Therefore, we will investigate further whether phaseolin affects the activity and recruitment of tumor-associated macrophages through NF-κB intervention in the tumor microenvironment.

Leukocyte migration, adhesion, and activation are the fundamental cellular changes during inflammatory processes, and are closely related to each other [40]. Therefore, effective control of these cellular changes is a major goal in the treatment of inflammatory diseases. For example, the development of novel therapeutic strategies that efficiently target the leukocyte adhesion may yield better results in future. A number of experimental studies suggest that Ninj1, an adhesion molecule, regulates macrophage migration, cell-to-cell/cell-to-matrix adhesion, and activation, and thus it can be used as a therapeutic target of diverse inflammatory diseases including experimental autoimmune encephalomyelitis [41,42], colitis [43], diabetes [44], and atherosclerosis [45]. Different approaches involving the usage of various neutralizing monoclonal antibodies and peptides for regulating the expression and action of Ninj1 have been reported. In addition, it could be a good strategy to identify bioactive ingredients which control the expression of Ninj1 from traditionally used natural products against inflammation. Our findings suggested that phaseolin, a constituent of Kushen with anti-inflammatory efficacy, exhibits a strong inhibitory effect on Ninj1 expression, which in turn is associated with reduced migration, adhesion, and activation of macrophages in vitro and in vivo. Recently it has been reported that Ninj1 is significantly upregulated in diabetes patients, and that functional blocking of Ninj1 attenuates IL-6 and MCP-1 overexpression and protects vascular defects [44]. This report was in the same context as where phaseolin exhibited concentration-dependent inhibition of the expression of IL-6 and MCP-1 increased by endotoxin (Figure 2). Thus, further studies are warranted to verify the pharmacological relevance of phaseolin in regulating the activation and recruitment of macrophages mediated by Ninj1 in inflammatory diseases such as diabetes.

In conclusion, our study identified the anti-inflammatory activities of phaseolin in Raw 264.7 cells through the downregulation of Ninj1 and NF-κB. Our findings have a broad application for cancers that exhibit highly activated NF-κB, such as breast cancers [39]. The potential benefit of phaseolin requires clinical investigation in patients with diabetes or atherosclerosis.

## Data Availability

Not applicable.
